# Chrysosplenol d, a Flavonol from *Artemisia annua*, Induces ERK1/2-Mediated Apoptosis in Triple Negative Human Breast Cancer Cells

**DOI:** 10.3390/ijms21114090

**Published:** 2020-06-08

**Authors:** Sophia J. Lang, Michael Schmiech, Susanne Hafner, Christian Paetz, Katharina Werner, Menna El Gaafary, Christoph Q. Schmidt, Tatiana Syrovets, Thomas Simmet

**Affiliations:** 1 Institute of Pharmacology of Natural Products and Clinical Pharmacology, Ulm University, 89081 Ulm, Germany; sophia.lang@uni-ulm.de (S.J.L.); michael.schmiech@uni-ulm.de (M.S.); susanne.hafner@uni-ulm.de (S.H.); katharina.werner@uni-ulm.de (K.W.); mennat_elgaafary@yahoo.com (M.E.G.); christoph.schmidt@uni-ulm.de (C.Q.S.); 2 Research Group Biosynthesis/Nuclear Magnetic Resonance, Max Planck Institute for Chemical Ecology, 07745 Jena, Germany; cpaetz@ice.mpg.de; 3 Department of Pharmacognosy, College of Pharmacy, Cairo University, Cairo 11562, Egypt

**Keywords:** chrysosplenol d, casticin, *artemisia annua*, mitogen-activated protein kinase, apoptosis, cell cycle, chick chorioallantoic membrane assay, methoxylated flavonoids, triple negative breast cancer

## Abstract

Triple negative human breast cancer (TNBC) is an aggressive cancer subtype with poor prognosis. Besides the better-known artemisinin, *Artemisia annua* L. contains numerous active compounds not well-studied yet. High-performance liquid chromatography coupled with diode-array and mass spectrometric detection (HPLC-DAD-MS) was used for the analysis of the most abundant compounds of an *Artemisia annua* extract exhibiting toxicity to MDA-MB-231 TNBC cells. Artemisinin, 6,7-dimethoxycoumarin, arteannuic acid were not toxic to any of the cancer cell lines tested. The flavonols chrysosplenol d and casticin selectively inhibited the viability of the TNBC cell lines, MDA-MB-231, CAL-51, CAL-148, as well as MCF7, A549, MIA PaCa-2, and PC-3. PC-3 prostate cancer cells exhibiting high basal protein kinase B (AKT) and no ERK1/2 activation were relatively resistant, whereas MDA-MB-231 cells with high basal ERK1/2 and low AKT activity were more sensitive to chrysosplenol d treatment. In vivo, chrysosplenol d and casticin inhibited MDA-MB-231 tumor growth on chick chorioallantoic membranes. Both compounds induced mitochondrial membrane potential loss and apoptosis. Chrysosplenol d activated ERK1/2, but not other kinases tested, increased cytosolic reactive oxygen species (ROS) and induced autophagy in MDA-MB-231 cells. Lysosomal aberrations and toxicity could be antagonized by ERK1/2 inhibition. The *Artemisia annua* flavonols chrysosplenol d and casticin merit exploration as potential anticancer therapeutics.

## 1. Introduction

Breast cancer is the second leading cause of cancer-related death in women in the United States. It is estimated that in 2020, 30% of all newly diagnosed cancers in females will be breast cancer [1]. Triple negative human breast cancer (TNBC) is an aggressive subtype of breast cancer, negative for estrogen and progesterone receptors, as well as for epidermal growth factor receptor 2. TNBC is usually diagnosed in females under the age of 50 with an incidence of 10–20% of all breast cancers. TNBC is therapy-resistant and is, therefore, characterized by poor prognosis. Non-halogenated anthracyclines and taxanes belong to the standard chemotherapeutic treatment regimen in breast patients including those with TNBC [2]. Unfortunately, at present, no approved targeted therapy is available for TNBC [3]. Therefore, novel agents for the prevention and treatment of breast cancer, particularly TNBC, are urgently required.

Plants and plant-derived compounds have a long-standing tradition of medicinal use. Even today, many cancer therapeutics are of natural origin as they are able to modulate pathways often deregulated in human cancers [4,5]. However, a vast number of diverse secondary metabolites of medicinal plants have not yet been comprehensively investigated [6]. Hence, natural compounds may still harbor new leads for the treatment of malignant diseases.

*Artemisia annua* L. is a medicinal plant used in traditional Chinese medicine for the treatment of fever. Currently, the sesquiterpene lactone artemisinin originally isolated from *Artemisia annua* is part of standard combination therapies to treat uncomplicated malaria [7]. Artemisinin and its derivatives contain an endoperoxide group, which in the presence of ferrous ion generates reactive oxygen species (ROS). Artemisinin derivatives exhibit antiparasitic, antimalarial, and anticancer activities that are augmented in the presence of iron complexes [8]. However, artemisinin and its derivatives are unstable leading to poor bioavailability [8]. On the other hand, *Artemisia annua* contains a variety of additional bioactive components worth to be investigated. Thus, the plant contains more than 50 different phenolic compounds (flavones, flavonols, coumarins, phenolic acids, etc.) making it one of the four medicinal plants with the highest oxygen radical absorbance capacity [8]. As the dietary consumption of flavonoids correlates inversely with cancer occurrence, it has been assumed that flavonoids might prevent, delay, or help to cure cancer by modulating oxidative stress associated with cancerogenesis [8]. In addition, *Artemisia annua* contains plenty of structurally diverse polymethoxylated flavonoids, which can increase bioavailability and enhance the therapeutic efficacy of artemisinin. Such methoxylated flavones are believed to be more stable and to possess better pharmacokinetic properties compared to hydroxylated flavonoids [8].

In the course of our investigations on antitumor efficacies of a number of commercially available *Artemisia annua* nutraceuticals, we have identified a commercial *Artemisia annua* extract (MoMundo GmbH, Bad Emstal, Germany) that exhibits potent cytotoxic activity in vitro [9]. Using fingerprint analysis and fractionation of the Momundo extract, we found that it does not contain any detectable artemisinin yet high amounts of the cytotoxic methoxylated flavonols, casticin and chrysosplenol d. Whilst some studies reported tubulin-binding and antiproliferative efficacy of casticin against breast, lung, and colon cancer cell lines [10,11], almost no information is available as to potential anticancer activities of chrysosplenol d [12]. Analysis of the structure-activity relationship of flavones revealed that the C2-C3 double bond, the C-3 hydroxyl- and the ortho-catechol moiety of ring B are important for high antiproliferative activity [8,13]. Since chrysosplenol d and casticin harbor several of these functionalities, the aim of the work was to analyze more closely their antiproliferative and apoptosis-inducing capacity in cancer cells in vitro and in vivo.

## 2. Results

### 2.1. Ingredients of the Momundo Artemisia Annua Dietary Supplement

**Figure 1:**
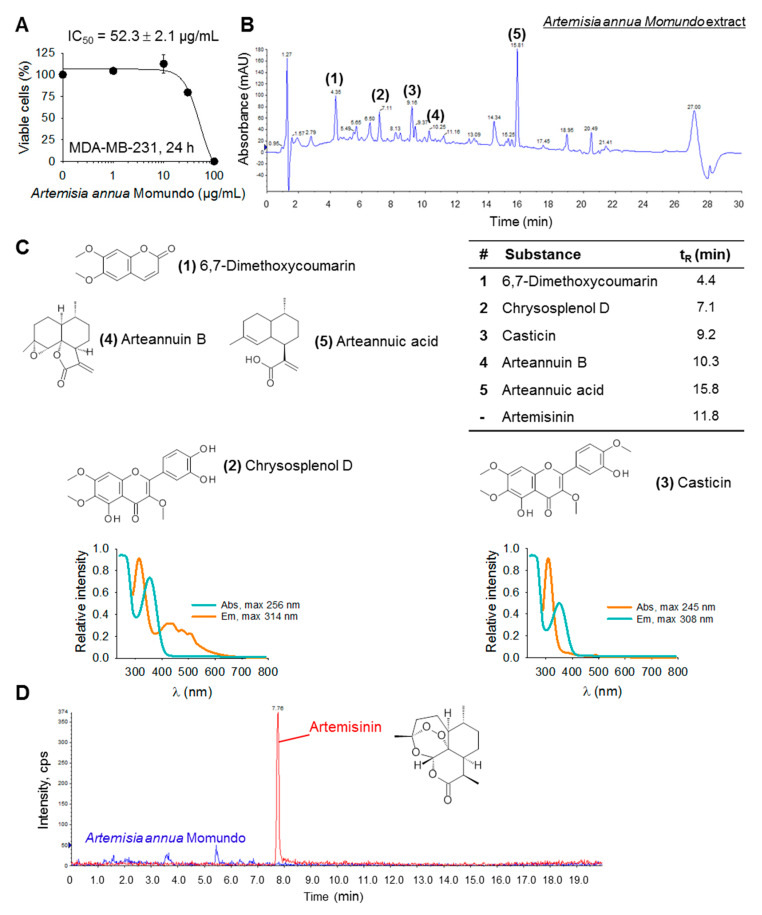
Most abundant compounds of an *Artemisia annua* dietary supplement. (**A**) Acetonitrile extract of the Momundo *Artemisia annua* dietary supplement is cytotoxic to MDA-MB-231 breast cancer cells as analyzed by 2,3-bis-(2-methoxy-4-nitro-5-sulfophenyl)-2h-tetrazolium-5-carboxanilide (XTT). (**B**) High-performance liquid chromatography coupled with diode-array and mass spectrometric detection (HPLC-DAD) fingerprint of the acetonitrile-enriched *Artemisia annua* Momundo extract. (**C**) The most abundant compounds were identified by comparison of retention times and mass spectra of reference substances or by ^1^H and ^13^C NMR spectroscopy. UV/Vis spectra of chrysosplenol d and casticin (methanol/water, 1:1) are shown. (**D**) HPLC-MS/MS chromatograms with multiple reaction monitoring (MRM) of artemisinin reference standard solution (red) and the Momundo extract (blue) indicating that the artemisinin concentration in the Momundo extracts is below the limit of detection (LOD = 0.2 ng/mg extract, recovery 94.8%).

For the identification of new compounds with anticancer properties in *Artemisia annua*, we analyzed an acetonitrile extract of the Momundo dietary supplement, which exhibited potent cytotoxic activity (Figure 1A). By HPLC-DAD-MS analysis and comparison with retention times and mass spectra of reference substances or by nuclear magnetic resonance (NMR) spectroscopy, the most abundant compounds in the Momundo *Artemisia annua* dietary supplement were identified as 6,7-dimethoxycoumarin, chrysosplenol d, casticin, arteannuin B, and arteannuic acid (Figure 1B,C). Of note, the extract contained no detectable artemisinin, with a detection limit of the quantification method of 0.2 ng/mg extract (Figure 1D). Subsequently, pure compounds were further investigated regarding their potential cytotoxic and antitumor efficacies using various treatment-resistant cancer cell lines.

### 2.2. Chrysosplenol d and Casticin Selectively Inhibit the Viability of Several Cancer Cell Lines

**Figure 2:**
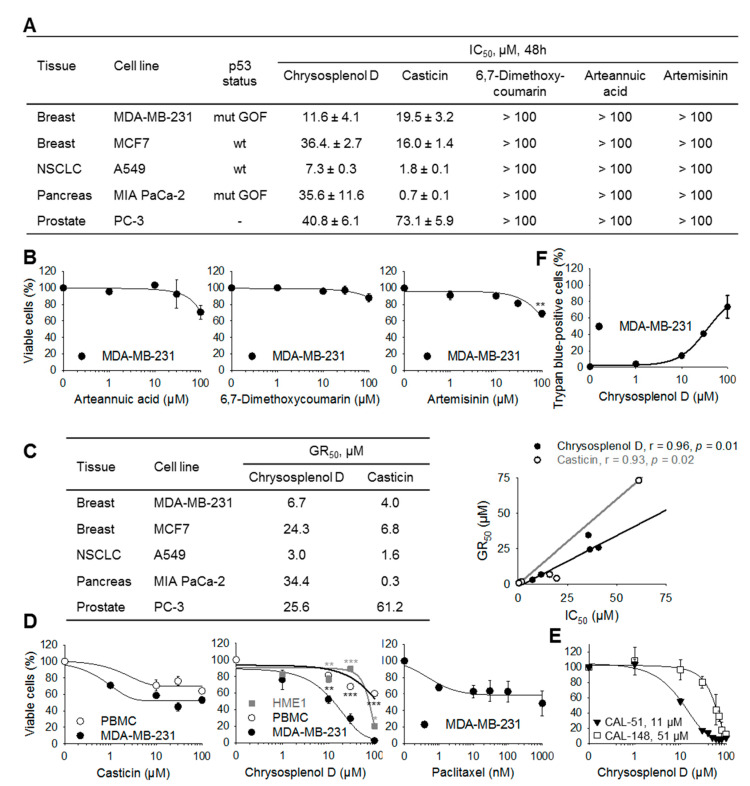
Chrysosplenol d and casticin, but not arteannuic acid, 6,7-dimethoxycoumarin, or artemisinin, inhibit selectively the viability of several cancer cell lines. Cells were treated with the various compounds for 48 h and analyzed by XTT (**A**-**E**). (**A**) NSCLC, non-small cell lung cancer; mut GOF, gain of function mutation. Apparent IC_50_ values for casticin are shown. (**B**) Viability of MBA-MB-231 cells after treatment with arteannuic acid, 6,7-dimethoxycoumarin, and artemisinin (** *p* < 0.01 vs. control). (**C**) Normalized growth rate inhibition values (GR_50_) and Pearson′s correlation between GR_50_ and IC_50_ values. (**D**) Peripheral blood mononuclear cells (PBMC, open black circles) and HME1 normal breast epithelial cells (closed grey squares) are relatively resistant to chrysosplenol d. Paclitaxel—positive control, * *p* < 0.05, ** *p* < 0.01, *** *p* < 0.001 vs. respective values of MDA-MB-231 cells. (**E**) Chrysosplenol d is toxic to the triple-negative breast cancer cell lines CAL-51 and CAL-148. Figures inside the graph are respective IC_50_ values. (**F**) Toxicity of chrysosplenol d to MDA-MB-231 cells was analyzed by trypan-blue exclusion after 48 h. Data are mean ± standard error of the mean (SEM) of *n* = 3–5.

Chrysosplenol d and casticin inhibited the viability of the MDA-MB-231 TNBC cells after 48 h with IC_50_ values of 11.6 and 19.5 µM, respectively. The most sensitive cell line towards chrysosplenol d and casticin was the non-small-cell lung carcinoma (NSCLC) cell line A549 and the most resistant one was the androgen-independent prostate carcinoma cell line PC-3. The hormone-sensitive breast cancer cells MCF7 exhibited higher resistance towards chrysosplenol d treatment compared to casticin. The pancreatic cancer cell line MIA PaCa-2 was particularly sensitive to casticin (IC_50_ = 0.7 µM), but less so to chrysosplenol d (IC_50_ = 36 µM) (Figure 2A). These results reveal that chrysosplenol d and casticin are cytotoxic for tumors of different origin although with different efficacies. Interestingly, within 48 h, 6,7-dimethoxycoumarin and arteannuic acid as well as artemisinin did not exhibit any significant antiproliferative effects on any of the cancer cell lines tested. Of note, artemisinin was not toxic to any of the cancer cell lines exhibiting IC_50_ > 100 µM to all those cell lines (Figure 2A,B).

Analysis of the toxicity of casticin and chrysosplenol d by using normalized growth inhibition (GR) values, which are independent from cell proliferation [14], revealed that chrysosplenol d exhibits a GR_50_ = 6.7 µM for MDA-MB-231. The GR_50_ values for chrysosplenol d and casticin correlate well with their respective IC_50_ values after 48 h of treatment (Figure 2C).

Analysis of the concentration-dependent effects on cancer cells revealed that casticin, similar to paclitaxel, is cytotoxic only to a proportion of cells (doubling time of MDA-MB-231 cells is about 30 h). In contrast, chrysosplenol d at high concentrations eventually eliminated all MDA-MB-231 cancer cells (Figure 2D) and those of two additional TNBC cell lines, CAL-51 and CAL-148 (Figure 2E). Notably, chrysosplenol d and casticin exhibited only little effects on the viability of peripheral blood mononuclear cells (PBMC). Likewise, HME1 normal breast epithelial cells were more resistant to chrysosplenol d treatment compared to breast cancer cells (Figure 2D). Similarly, analysis of dead cells by trypan-blue exclusion assay demonstrated cytotoxicity of chrysosplenol d to MDA-MB-231 cells (Figure 2F). Luciferase-based assays were not used, because chrysosplenol d inhibited the NanoLuc enzyme (Promega) with an IC_50_ = 8.7 ± 0.6 µM.

### 2.3. Chrysosplenol d and Casticin Inhibit the Cell Cycle Progression of MDA-MB-231 Cells in Vitro

**Figure 3:**
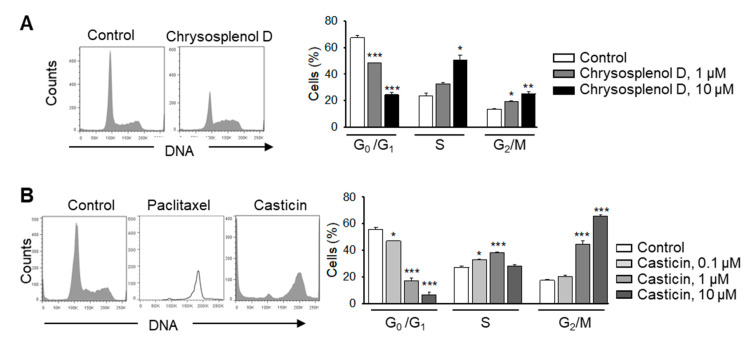
Chrysosplenol d and casticin induce cell-cycle aberrations in breast cancer cells. (**A**) MDA-MB-231 cells were treated with different concentrations of chrysosplenol d for 48 h, stained with propidium iodide, and analyzed by using flow cytometry. Representative histograms after treatment with chrysosplenol d (1 µM) are shown (left hand panel). Graph shows statistical analysis (right hand panel) (**B**) Cells were treated with different concentrations of casticin for 48 h and were analyzed as in (**A**). Concentrations were chosen on the basis of XTT assays showing cytotoxicity of casticin already at 1 µM. Representative histograms after treatment with casticin (1 µM, 48 h) and paclitaxel (100 nM, 24 h) are shown (left hand panel). Graph shows statistical analysis (right hand panel). Data are mean ± SEM, *n* = 3, * *p* < 0.05, ** *p* < 0.01, *** *p* < 0.001 vs. controls.

Chrysosplenol d induced cell cycle aberrations with accumulation of cells in the S-phase and partially in the G_2_/M-phase of the cell cycle, whereas the amount of cells in the G_1_-phase was concentration-dependently reduced (Figure 3A). This might indicate that cells in the G_2_/M-phase are particularly sensitive to chrysosplenol d and that they might undergo apoptotic cell death mimicking the DNA content of cells in the S-phase. Differently, and similar to paclitaxel, casticin induced a concentration-dependent arrest of the majority of cells in the G_2_/M-phase and a reduction of cells in the G_1_-phase (Figure 3B).

### 2.4. Chrysosplenol d and Casticin Induce Apoptosis in Breast Cancer Cells

**Figure 4:**
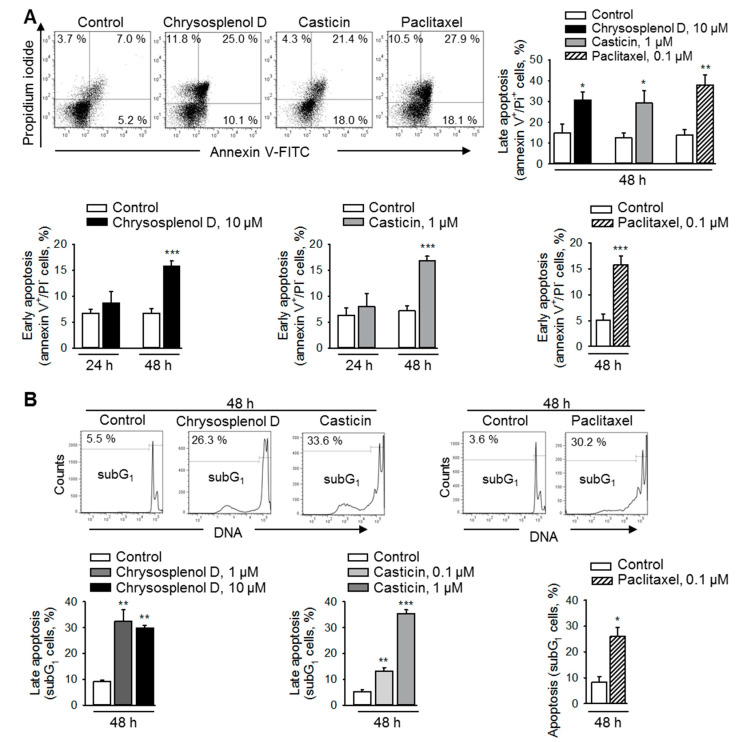
Chrysosplenol d and casticin induce apoptosis in breast cancer cells. (**A**) MDA-MB-231 cells were treated with chrysosplenol d, casticin, or paclitaxel (100 nM), stained with annexin V- fluorescein isothiocyante (FITC)/propidium iodide, and analyzed by flow cytometry. Early apoptotic apoptotic cells (annexin V+/PI- cells) and late apoptotic cells (annexin V+/PI+) are shown. Representative dot blots after 48 h of treatment with chrysosplenol d (10 µM), casticin (1 µM), or paclitaxel (100 nM) are shown, *n* = 3–5. (**B**) Cells were treated as described in (**A**). DNA was stained with propidium iodide and analyzed by using flow cytometry, *n* = 3. Data are mean ± SEM, * *p* < 0.05, ** *p* < 0.01, *** *p* < 0.001 vs. controls.

Phosphatidylserine exposure, an early sign of apoptosis [15], was analyzed by annexin-V/propidium-iodide double staining. Cells treated with chrysosplenol d, casticin, or paclitaxel for 48 h exhibited increased numbers of annexin-V-positive and propidium-iodide-negative cells characteristic for early apoptotic cells (Figure 4A). A further, highly distinct late apoptotic feature is DNA condensation, fragmentation of the nucleus, and formation of apoptotic bodies [16]. Similar to paclitaxel, both compounds, chrysosplenol d and casticin, significantly increased the number of cells with hypodiploid DNA contents demonstrating DNA fragmentation and formation of apoptotic bodies (Figure 4B). The number of cells with fragmented DNA (Figure 4B) after treatment with chrysosplenol d and casticin D correlates well with the number of late apoptotic, double annexin-V/propidium-iodide-positive cells (Figure 4A).

### 2.5. Chrysosplenol d and Casticin Inhibit Tumor Growth of Breast Cancer Xenografts in Vivo

**Figure 5:**
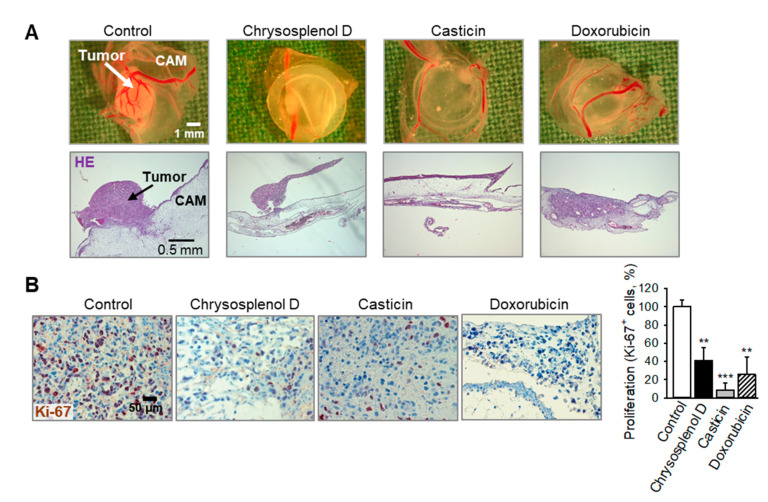
Chrysosplenol d and casticin inhibit proliferation of breast cancer xenografts in vivo. MDA-MB-231 xenografts grown on chick chorioallantoic membranes (CAM) were treated for 3 d with either chrysosplenol d, casticin (each at 30 µM), or doxorubicin (1 µM) as positive control. On day 4 after initiation of treatment, tumors were collected and analyzed immunohistochemically. (**A**) Representative pictures of tumor xenografts immediately after extraction (upper row) or hematoxylin and eosin (HE) staining (lower row, original magnification 50×). (**B**) Immunohistochemical staining of proliferating Ki-67+ cells (brown nuclear stain). Nuclei were counterstained with hematoxylin (blue). Original magnification 200×. Graph shows reduction of proliferating Ki-67+ cells compared to controls. Data are mean ± SEM, *n* = 4–7, ** *p* < 0.01, *** *p* < 0.001 vs. control.

For verification of an antitumor activity of chrysosplenol d and casticin in vivo, MDA-MB-231 breast cancer xenografts grown on the chorioallantoic membrane (CAM) of fertilized chick eggs [17] were treated with chrysosplenol d, casticin, or the anthracycline doxorubicin, an anticancer drug used in most chemotherapeutic combination regimens for breast cancer [3]. Chrysosplenol d and casticin reduced the tumor mass to a similar extent as the standard chemotherapeutic doxorubicin (Figure 5A). Immunohistochemistry of the xenografts demonstrated a significantly reduced expression of the proliferation antigen Ki-67 (Figure 5B) revealing growth inhibition of solid tumors in vivo. Notably, chick embryos treated with chrysosplenol d or casticin did not show any signs of systemic toxicity.

### 2.6. Chrysosplenol d and Casticin Induce Mitochondrial Membrane Permeabilization in Breast Cancer Cells

**Figure 6:**
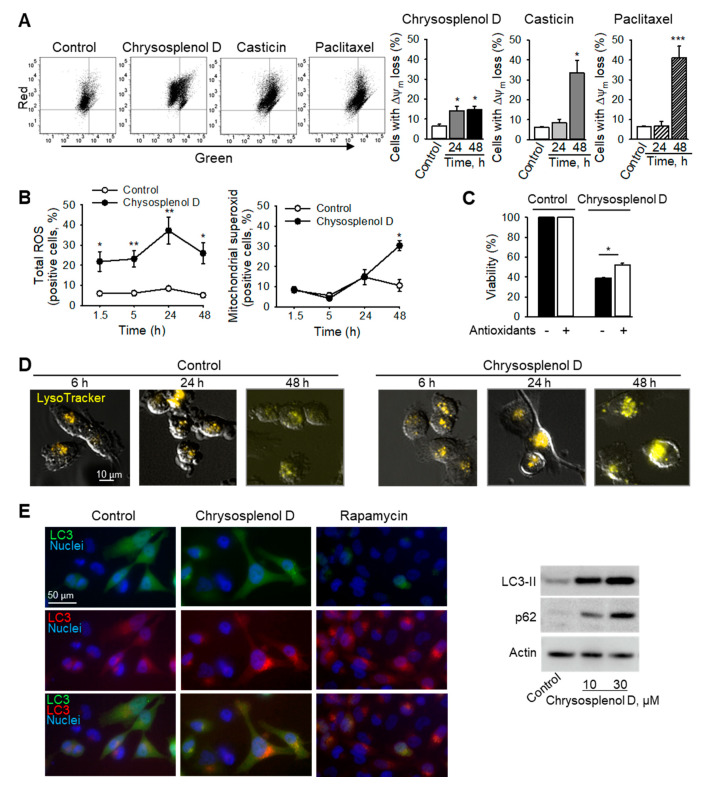
Chrysosplenol d induces ROS, causes dissipation of the mitochondrial membrane potential (ΔΨ_m_), and increases number and size of acidic organelles in breast cancer cells. (**A**) Loss of mitochondrial membrane potential in MDA-MB-231 cells after treatment with either chrysosplenol d (10 µM), casticin (1 µM), or paclitaxel (100 nM) was analyzed by using flow cytometry after staining with JC-1. Representative dot plots after 48 h are shown (left hand panels). Graphs show statistical analyses (right hand panels). (**B**) Total and mitochondrial ROS were analyzed by using flow cytometry in MDA-MB-231 cells treated with chrysosplenol d (30 µM) with H_2_DCFDA and MitoSOX™ Red, respectively. (**C**) ROS scavengers attenuate the chrysosplenol d-induced toxicity. MDA-MB-231 were pretreated with a mixture of Trolox (100 µM) and mitoTEMPO (10 µM) for 1 h and then treated with chrysosplenol d (10 µM) for 48 h. Cell viability was analyzed by XTT. (**D**) MDA-MB-231 cells were treated with chrysosplenol d (10 µM), stained with LysoTracker and analyzed microscopically. Original magnification 200×. (**E**) Analysis of autophagy. GFP-RFP-LC3 HeLa cells were treated for 4 h and analyzed as in (**D**). Rapamycin (1 µM)—positive control. Western immunoblot shows induction of autophagy in MDA-MB-231 cells treated for 24 h. Data are mean ± SEM, *n* = 3–4, * *p* < 0.05, ** *p* < 0.01, *** *p* < 0.001 vs. controls.

Most signals leading to apoptosis are orchestrated by mitochondria. Mitochondria contain a variety of proapoptotic molecules capable of executing apoptosis once the outer mitochondrial membrane is permeabilized [16]. An early sign of mitochondrial damage is loss of mitochondrial membrane potential (ΔΨ_m_) [18]. Treatment with chrysosplenol d significantly increased the number of cells with ΔΨ_m_ loss already after 24 h. Similar to paclitaxel, casticin induced loss of ΔΨ_m_, but only after 48 h (Figure 6A). However, although chrysosplenol d increased the total level of reactive oxygen species (ROS) already within 90 min of treatment, the level of mitochondrial superoxide increased only after 48 h (Figure 6B). The ROS scavengers, Trolox and mitoTEMPO, attenuated the chrysosplenol d-induced cytotoxicity (Figure 6C). Interestingly, chrysosplenol d-treated cells exhibited an early and prolonged increase in the size and number of lysosomes (Figure 6D). Similarly, chrysosplenol d induced accumulation of LC3II and p62 autophagic markers in MDA-MB-231 cells and formation of fluorescent LC3-positive autophagosomes in GFP-RFP-LC3-expressing HeLa cells (Figure 6E). The GFP fluorescence is more sensitive to acidic pH compared to RFP. Hence, the fact that the number of red puncta exceeded that of green indicated that chrysosplenol d did not inhibit the autophagic flux, the fusion of autophagosomes with lysosomes or the degradation of the autophagosomal cargo.

### 2.7. Chrysosplenol d Activates ERK1/2

**Figure 7:**
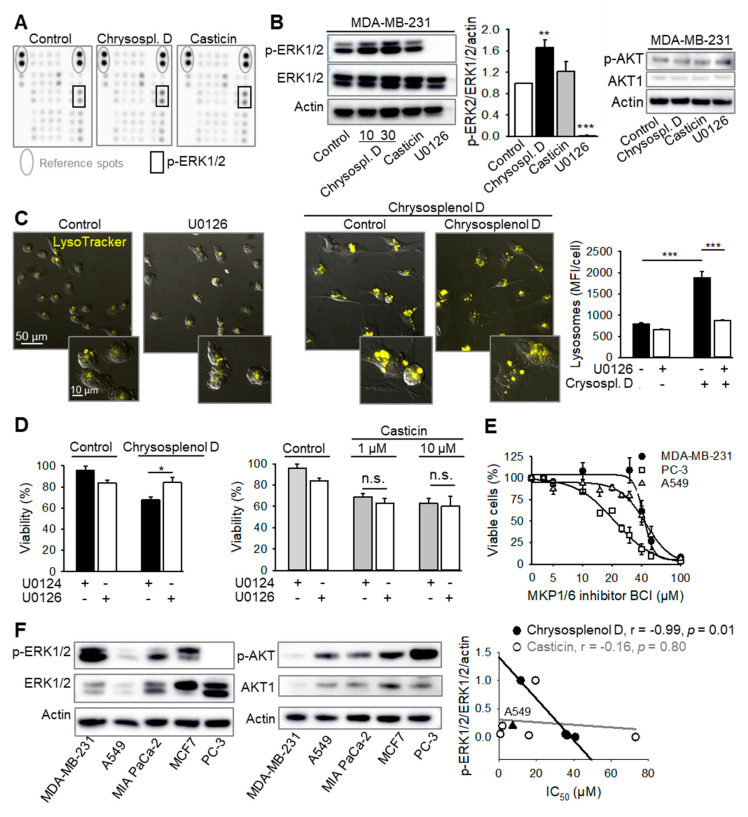
Chrysosplenol d and casticin induce sustained increase of the basal ERK1/2 activation in breast cancer cells. (**A**) MDA-MB-231 cells were treated with the compounds (30 µM) for 3 h and cell lysates were analyzed by using phospho-kinase protein array. Altogether 43 different kinases were analyzed. Representative membranes from 2 experiments are shown. (**B**) ERK1/2 phosphorylation was confirmed by western immunoblotting, whereas neither compound induced AKT phosphorylation in MDA-MB-231 cells. MDA-MB-231 cells were treated with chrysosplenol d (10–30 µM), casticin (1 µM), or U0126 (5 µM) for 3 h, and analyzed by western immunoblotting with antibodies against p-ERK1/2, ERK1/2, p-AKT (S473), and AKT1. Actin–loading control. Representative blots and quantification (chrysosplenol d, 10 µM) are shown. (**C**) Pretreatment with the MEK-inhibitor U0126 (5 µM, 1 h) blocked lysosomal aberrations induced by chrysosplenol d (10 µM, 48 h). Cells were stained with LysoTracker and analyzed microscopically, magnification 200×. Graph shows quantification of 10 high power fields each from 3 independent experiments. (**D**) Pretreatment with the MEK-inhibitor U0126, but not the inactive analogue U0124 (both at 5 µM, 1 h), attenuated the toxicity of chrysosplenol d (10 µM), but not that of casticin (1–10 µM). Cell viability was analyzed after 48 h by XTT. (**E**) Inhibition of ERK1/2 dephosphorylation leading to prolonged ERK1/2 activation is toxic to cancer cells. Cells were treated with the dual MKP1/6 inhibitor BCI for 24 h and analyzed by using XTT. (**F**) ERK1/2 and AKT activation pattern in different cancer cell lines. Whole cell extracts were analyzed as in (B); representative blots are shown. IC_50_ values of chrysosplenol d (closed symbols) in MDA-MB-231, MIA PaCa-2, MCF7 and PC-3 cells, but not of casticin (open symbols), treated with chrysosplenol d, inversely correlate with basal ERK1/2 activity analyzed by western immunoblotting, Pearson′s correlation; A549—outliner. Data are mean ± SEM, *n* = 3, * *p* < 0.05, ** *p* < 0.01, *** *p* < 0.001 vs. respective controls.

To identify potential targets of chrysosplenol d and casticin, the phosphorylation profiles of different kinases and respective protein substrates were analyzed by a human phospho-kinase array. Chrysosplenol d and partially casticin increased the basal ERK1/2 activation by phosphorylation on threonine-202/tyrosine-204 and threonine-185/tyrosine-187 after 3 h of treatment, but had no effect on the other kinases analyzed (Figure 7A). These results were further confirmed by western immunoblotting in MDA-MB-231 treated with 10–30 µM of chrysosplenol d and 1 µM casticin for 3 h (Figure 7B). Thus, chrysosplenol d and, to a lesser extent, casticin increased the already high basal ERK1/2 activation in breast cancer cells. However, neither chrysosplenol d nor casticin affected AKT phosphorylation in breast cancer cells at this time point (Figure 7B) and inhibition of AKT activation did not affect the chrysosplenol d-induced cytotoxicity (Figure S3).

To establish the role of ERK1/2 activation in cell death induced by chrysosplenol d and casticin, MEK kinases upstream of ERK1/2 were inhibited by U0126. ERK1/2 inhibition by U0126 blocked the chrysosplenol d-induced increase of lysosomal size (Figure 7C), but it had no effect on chrysosplenol d-induced loss of mitochondrial membrane potential (ΔΨ_m_). Moreover, treatment with U0126 alone induced ΔΨ_m_ dissipation (Figure S4).

MEK/ERK1/2 inhibition did not rescue cells from casticin-induced toxicity. Differently, chrysosplenol d-induced cell death was strongly attenuated by MEK/ERK1/2 inhibition with U0126, but not with U0124, a structurally similar control compound that does not inhibit MEK [19] (Figure 7D). Similarly, ERK activation by (E)-2-benzylidene-3-(cyclohexylamino)-2,3-dihydro-1H-inden-1-one (BCI), a dual MKP1/6 phosphatase inhibitor that targets exclusively ERK1/2 [20,21], also induced concentration-dependent toxicity in different cancer cell lines including MDA-MB-231 (Figure 7E).

Remarkably, the basal ERK1/2 activity in MDA-MB-231, MCF7, MIA PaCa-2, and PC-3, but not in A549 cells, inversely correlates to the IC_50_ values of chrysosplenol d, but not to those of casticin, in these cells (Figure 7F). The chrysosplenol d-resistant PC-3 cells exhibit no basal ERK1/2 activation but high activation of the phosphoinositide 3-kinase (PI3K)/AKT axis (Figure 7F). Likewise, the relatively resistant MCF7 cells exhibit higher AKT activation, whereas MDA-MB-231 and A549 cells, which are more sensitive to chrysosplenol d, exhibit low PI3K/AKT activity (Figure 2 and Figure 7F). Thus, the AKT activation pattern in the cell lines analyzed correlates inversely with the cell sensitivity to chrysosplenol d, i.e., higher PI3K/AKT activity is associated with higher cell resistance.

## 3. Discussion

The present study provides evidence that the two *Artemisia annua* flavonols, chrysosplenol d and casticin, might be promising antitumor compounds with different mechanisms of action. The differential cytotoxicity to cancer cells with much lower cytotoxicity to PBMC and normal breast epithelial cells and no overt systemic toxicity to chick embryos point to selectivity of the flavonols for cancer cells and implicate low systemic toxicity in vivo. These findings encouraged us to further analyze potential molecular mechanisms of action of chrysosplenol d and casticin.

Uncontrolled proliferation caused by deregulated cell cycle control mechanisms is a common feature of neoplasia and many cancers are reported to be selectively vulnerable to the inhibition of cell cycle regulatory proteins [22,23]. Hence, targeting the cell cycle is an attractive feature for an anticancer drug. Both compounds induced cell cycle aberrations. However, chrysosplenol d increased the number of cells in the S- and G_2_/M-phases, whereas casticin strongly increased the number of cells in the G_2_/M-phase only. Analysis of cell viability demonstrated, that even relatively high concentrations of casticin, similar to paclitaxel, are cytotoxic only to a proportion of cells, whereas chrysosplenol d eventually eliminated all cancer cells. These findings are consistent with a study reporting casticin to bind to tubulin and to induce G_2_/M-phase arrest in MCF7 breast cancer and H1299 lung carcinoma cell lines [10]. Hence, the data indicate that despite structural similarities, chrysosplenol d and casticin target different signaling pathways in cancer cells.

Induction of apoptosis in cancer cells by a chemotherapeutic drug is an advantageous feature, because apoptosis is a highly regulated process that does not induce inflammation and does not negatively affect function of neighboring non-transformed cells [15]. During early apoptosis, phosphatidylserine becomes exposed on the outer leaflet of the plasma membrane representing an initial signal for engulfment by phagocytes [15]. This observation, together with the occurring later DNA-fragmentation, reveals that the *Artemisia annua* flavonols chrysosplenol d and casticin induce apoptosis in treatment-resistant MDA-MB-231 TNBC cells.

Permeabilization of the outer mitochondrial membrane is a central step in induction of intrinsic apoptosis [16]. In casticin- as well as in paclitaxel-treated cells, mitochondrial damage did not precede the induction of apoptosis, but occurred at the same time as phosphatidylserine exposure. These data are in agreement with the finding that casticin and paclitaxel as tubulin binding agents do not primarily target mitochondria in cancer cells. In contrast, in chrysosplenol d-treated cells, dissemination of the mitochondrial membrane potential took place 24 h earlier than any sign of apoptosis could be detected. This finding might suggest an active role of mitochondria in the induction of apoptosis by chrysosplenol d. However, an increase in mitochondrial superoxide occurred quite late, at the time when also apoptosis signs have been detected. Differently, total ROS levels were increased quite early and addition of ROS scavengers attenuated chrysosplenol d-induced toxicity.

Early ROS induction may promote sustained ERK activation [20]. MDA-MB-231 cells as used in our study express mutated K-ras [24] and exhibit already significantly activated basal ERK1/2 phosphorylation. Still, treatment with chrysosplenol d increased the basal ERK1/2 phosphorylation. Also, the flavonol quercetin, which is structurally similar to chrysosplenol d, was shown to induce activation of ERK1/2 [25]. Different to MDA-MB-231, PC-3 cells are relatively resistant to chrysosplenol d. PC-3 lack the PTEN phosphatase, which results in aberrant activation of the phosphoinositide 3-kinase (PI3K)/AKT pathway and no basal ERK1/2 activation as demonstrated by us and others [26,27,28]. The mechanisms of the antagonistic relationship between ERK1/2 and AKT might involve a negative ERK1/2 feedback signaling affecting upstream Ras/ERK1/2 activators including prosurvival signaling of the phosphoinositide 3-kinase/AKT/mTOR [29]. Hence, high basal ERK1/2 and low basal AKT activity might be indicative for the sensitivity of cells to chrysosplenol d.

ERK1/2 are part of the pro-oncogenic Ras/Raf/MEK signaling pathway, which is deregulated in numerous cancers due to frequent activating mutations in Ras and B-Raf genes [30]. Hence, ERK1/2, kinases downstream of Ras/Raf/MEK, are often activated in cancer promoting cell proliferation and resistance to apoptosis. Thus, basal ERK1/2 activation is necessary for ΔΨ_m_ maintenance probably through activation of prosurvival BCL2 proteins [31]. Accordingly, treatment with the MEK-inhibitor U0126 induced ΔΨ_m_ dissipation in MDA-MB-231 cells.

Alternatively and paradoxically, under certain conditions, upregulated ERK1/2 can also induce apoptosis, autophagy, or senescence [32]. Thus, similar to chrysosplenol d, ERK activation by BCI, a dual MKP1/6 phosphatase inhibitor, which targets exclusively ERK1/2 [20,21], induced concentration-dependent toxicity in different cancer cell lines including MDA-MB-231. Hence, an enhanced and sustained ERK1/2 activation may trigger proapoptotic effects.

There are several hypothesis how sustained ERK1/2 activation might promote apoptosis. Thus, ERK1/2 might induce expression of proapoptotic genes or directly activate caspase 8, albeit also through *de novo* protein synthesis [20]. In our experiments, ERK1/2 inhibition preceded treatment with chrysosplenol d by 1 h, which might not suffice for new protein synthesis [26]. Likewise, ERK1/2 activity has been demonstrated to directly target mitochondrial respiration, to promote disruption of mitochondrial membrane potential (ΔΨ_m_), to induce membrane permeability, and to induce the release of cytochrome c and other proapoptotic proteins. Furthermore, phosphorylated ERK1/2 has been localized on mitochondrial membranes [20]. Our data, however, demonstrate a rather late increase in mitochondrial peroxide levels indicating that mitochondria might not be the primary target of chrysosplenol d.

ERK1/2 can also bind to and stabilize p53 in different ways, thus augmenting p53 expression, which is required for the ERK1/2-induced apoptosis under certain conditions [20]. In line with that, p53-deficient PC-3 cells are also resistant to treatment with chrysosplenol d [33], whereas NSCLC A549 expressing wild type p53 (p53^wt^) are particularly sensitive to chrysosplenol d. However, the p53 status alone does not suffice to explain the differential sensitivity of cancer cell types to chrysosplenol d. Thus, MCF7 breast cells are also p53^wt^ and are relatively resistant to chrysosplenol d, whilst MDA-MB-231, which express gain of function p53^mut^ that promotes tumor growth independent from classical downstream targets of p53 [33,34], are relatively sensitive to treatment. Also, MIA PaCa-2 harbor a gain of function mutation of p53 [35], but are relatively resistant to chrysosplenol d treatment. Hence, although the p53 expression status might contribute to apoptosis induction by chrysosplenol d and casticin in some cancer cell types and should be considered, activation of other pathways should not be neglected.

Sustained ERK activation can induce autophagy [20]. Indeed, cells treated with chrysosplenol d exhibited increased prolonged ERK1/2 activation and an increased number and size of lysosomes indicating early induction of autophagy. Autophagy, though generally considered as a prosurvival process, might as well promote apoptosis [36]. Remarkably, the ability of ERK1/2 to activate autophagy and proapoptotic pathways depends on the strength of their activation. Prolonged aberrant signaling through ERK kinases leads to a proteasome-dependent degradation of multiple phosphoproteins required for cell growth and cell-cycle progression and it is characterized by mitochondrial dysfunction. It has been suggested that such aberrant ERK1/2 activities are recognized by intracellular tumor-suppressor pathways, which promote apoptosis [29]. This scenario is in agreement with the effects induced by chrysosplenol d in cancer cells, i.e., increased ERK activation, increased formation of acidic organelles, and cytotoxicity with signs of both, apoptotic and autophagic cell death. In addition, both, chrysosplenol d-induced toxicity and lysosomal aberrations were strongly attenuated by MEK/ERK1/2 inhibition. Differently, casticin-induced toxicity was not antagonized by U0126.

In summary, the data presented suggest that the *Artemisia annua* flavonols chrysosplenol d and casticin are potential antitumor compounds with different mechanism of action. Unlike the toxicity of casticin, the one of chrysosplenol d on cancer cells depends on sustained increased ERK1/2 activation.

## 4. Materials and Methods

### 4.1. General Experimental Procedures

Momundo *Artemisia annua* extract was obtained from MoMundo GmbH (Bad Emstal, Germany). Arteannuin B and arteannuic acid were from Carbosynth (Berkshire, UK), casticin and 6,7-dimethoxycoumarin from Extrasynthese (Genay cedex, France), chrysosplenol d from ChemFaces (Wuhan, Hubei, China), and artemisinin from Sigma (St. Louis, MO, USA). Stock solutions were prepared in dimethyl sulfoxide (DMSO) and further diluted with appropriate medium supplemented with 1% heat-inactivated fetal bovine serum (FBS) directly before the experiments. The final DMSO concentration in the medium was 0.5%. Propidium iodide, dual specificity protein phosphatase 1/6 inhibitor (E)-2-benzylidene-3-(cyclohexylamino)-2,3-dihydro-1H-inden-1-one (BCI), DNase-free RNase A, and paclitaxel were from Sigma. The XTT (2,3-bis-(2-methoxy-4-nitro-5-sulfophenyl)-2h-tetrazolium-5-carboxanilide) cell proliferation assay was purchased from Roche Diagnostics (Filderstadt, Germany). The mitochondrial potential sensor JC-1, H_2_DCFDA, MitoSOX™ Red, LysoTracker Red were from Molecular Probes (San Diego, CA, USA). FITC-labeled annexin V and phenol-free matrigel were purchased from BD Biosciences (Heidelberg, Germany).

### 4.2. Analytical Fingerprint of Momundo Artemisia annua Dietary Supplement by HPLC-DAD-MS Analysis

HPLC-MS analysis was performed on an Agilent 1260 Infinity system (Agilent, Santa Clara, CA) coupled with an AB API 2000 (Applied Biosystems, Foster City, CA, USA) triple quadrupole mass spectrometer via an electrospray ionization source (ESI). The data were obtained and processed through Analyst 1.6.1 software (Ab Sciex, Framingham, MA, USA).

Chromatographic separation was achieved using an analytical HPLC column (ReproSil-Pur Basic C18-HD, 3 µm, 125 × 3 mm, Dr. Maisch, Ammerbuch-Entringen, Germany) with a precolumn (ReproSil-Pur Universal RP, 5 µm, 10 × 4 mm, Dr. Maisch). The flow rate was 600 µL/min and the injection volume was 5 µL. The mobile phase consisted of eluent A (deionized, ultrapure water + 0.05% formic acid) and eluent B (acetonitrile + 0.05% formic acid). Initial conditions were 70% eluent A and 30% eluent B followed by a linear gradient to 95% eluent B over 18 min, then 95% eluent B until 24 min. Thereafter, followed a linear gradient to initial conditions until 25 min and reequilibration for additional 5 min. To stabilize the chromatographic system, the column was kept at 28 °C. The eluent was scanned with a photodiode array detector at 210 nm, 254 nm, and 280 nm. MS detection was accomplished in positive and negative atmospheric pressure electrospray ionization (ESI) modes, and in single quadrupole scan mode. The substances were identified by comparison of retention times and mass spectra with reference compounds. Chrysosplenol d was subjected additionally to ^1^H and ^13^C NMR spectroscopy on a Bruker DRX 500 NMR spectrometer (Figures S1 and S2).

### 4.3. Quantification of Artemisinin by HPLC-MS/MS

The HPLC system used including columns and the mass spectrometer are described above. For sample preparation, 30 mg of Momundo capsule content were dissolved in 1.5 mL acetonitrile and extracted for 1 h at RT with continuous stirring. After centrifugation (16,000 g, 10 min), 1 mL supernatant was added to 1 mL water and filtered through regenerated cellulose (0.45 µm). The resulting solution with a sample concentration of 10 mg/mL was analyzed in triplicates. For chromatographic separation, the flow rate was set to 600 µL/min and the injection volume to 70 µL. The mobile phase consisted of eluent A (deionized, ultrapure water + 0.1% acetic acid and 10 mM ammonium acetate) and eluent B (acetonitrile + 0.1% acetic acid). Initial conditions were 60% eluent A and 40% eluent B followed by a linear gradient to 90% eluent B over 10 min, then 90% eluent B until 13 min. Thereafter, a linear gradient continued to initial conditions until 15 min and reequilibration until 20 min. The column was kept constantly at 28 °C.

MS/MS analysis was performed in the positive atmospheric pressure ESI and multiple-reaction monitoring (MRM) detection modes. For quantification, the precursor ion at m/z 300.2 ([M + NH_4_]^+^) and the product ion of highest intensity at m/z 151.2 were selected. To achieve linearity and to determine the limit of detection (LOD) and the limit of quantification (LOQ), standard solutions in the range from 7.5 ng/mL to 100 ng/mL (6 levels) were analyzed, each in triplicates, yielding a LOD of 2 ng/mL and a LOQ of 8 ng/mL. To evaluate accuracy, the recovery was determined by using the standard addition method. Hence, a real sample was spiked at six levels, extracted as described above in sample preparation and analyzed, each in triplicates, yielding a recovery of 94.8% (± 9.6% SD). Precision was determined by analysis of a reference sample with six replicates at four different days yielding the intraday variation of 1.5% (RSD) and the interday variation of 1.8% (RSD). Sample analysis showed that the artemisinin concentration of the Momundo extract was below the LOD. The method was also used for analysis of three additional commercial *Artemisia annua* herbal preparations. The analysis revealed artemisinin contents below the LOQ in ‘Artemisinin′ (Euro Nutrador B.V., Landgraaf, Netherlands) and ‘Artemisia Extrakt 400 mg′ (Vita-World, Taunusstein, Germany), whereas ‘Artemisia annua intense^®^ 600 mg′ (Novofrom Pharma GmbH, Gaggenau, Germany) contained 38 µg/mg artemisinin, which complies with the manufacturers specification of 4% artemisinin.

### 4.4. Cell Culture

Cell lines derived from advanced, therapy-resistant human tumors were obtained from the American Type Culture Collection (ATCC, Rockville, MD, USA) or the DSMZ-German Collection of Microorganisms and Cell Cultures (Braunschweig, Germany). HeLa-Difluo™ hLC3 autophagy reporter cells were from Invivogen (San Diego, CA, USA). Cells were cultured according to the supplier′s recommendations. PBMC were isolated from whole blood of healthy donors by density gradient centrifugation using Biocoll separating solution (Biochrom GmbH, Berlin, Germany) as previously described [37,38]. The collection and analysis of peripheral blood mononuclear cells used in this study was approved by the institutional Ethics Committee (# 177/18). The participating volunteers provided written informed consent to participate in this study.

### 4.5. Analysis of Cell Viability

Cell viability was analyzed by a XTT assay (Roche Diagnostics) according to the manufacturer′s instructions. Cells were seeded in 96-well plates and were treated with the Momundo *Artemisia annua* extract or compounds for 48 h. For some experiments, cells were pretreated with 5 µM of the MEK inhibitor U0126 (Biomol, Hamburg, Germany) or its inactive analogue U0124 (Bio-Techne, Minneapolis, MN, USA) for 1 h followed by incubation with chrysosplenol d or casticin (each 10 µM) for 48 h. Final DMSO concentrations did not exceed 0.5%. Absorbance of the orange formazan salt formed by mitochondrial reduction of the tetrazolium salt by viable cells was measured using an Tecan Infinite M1000 PRO plate reader (Männedorf, Switzerland) at 450 nm with a 630 nm reference filter.

### 4.6. Cell-Cycle Analysis

Cells were treated with different concentrations of the indicated compounds for 48 h. Then, cells were fixed with ice-cold 70% ethanol overnight. DNA was stained with propidium iodide in a buffer containing RNase for 1 h. Cells were analyzed by flow cytometry using a BD FACSVerse flow cytometer (BD Biosciences, San Jose, CA, USA). Cell-cycle analysis was performed with FlowJo software (TreeStar Inc., Ashland, OR, USA).

### 4.7. Breast Cancer Xenografts

For analysis of tumor growth in vivo [17,39], 0.75 × 10^6^ MDA-MB-231 cells were xenografted onto the chick chorioallantoic membrane (CAM) in medium/matrigel (1:1, *v*/*v*) 7 days after fertilization [40]. Starting from day 1 after seeding, cells were treated topically for 3 consecutive days with either 20 µL of the compounds, doxorubicin, or 0.5% DMSO in 0.9% NaCl. Due to lower embryonic toxicity, doxorubicin instead of paclitaxel was chosen as positive control. On day 4 after initiation of the treatment, tumors were collected, fixed, and embedded in paraffin. Five µm-sections were stained with hematoxylin, eosin, or were further analyzed using antibodies against the nuclear proliferation marker Ki-67 (M7240, Dako, Glostrup, Denmark). Images were recorded with an Axio Lab.A1 microscope (Carl Zeiss, Göttingen, Germany) and a Zeiss 2/3” CMOS-camera using Progres Gryphax software (Jenoptik, Jena, Germany).

### 4.8. Analysis of Apoptosis

Early apoptotic cells were analyzed flow cytometrically by annexin V and propidium iodide double staining. Briefly, cells were treated with the compounds or paclitaxel for 24 and 48 h, harvested by trypsination, and incubated for 15 min in full growth medium for membrane regeneration at 37 °C. Cells were stained with fluorescein isothiocyante (FITC)-labeled annexin V in a buffer containing calcium for 30 min and propidium iodide was added 1 min before measurement. The percentage of cells with subdiploidal DNA content was analyzed by flow cytometry. After treatment with the compounds or paclitaxel for 48 h, DNA was stained with propidium iodide using the same protocol as described for cell cycle analysis [27].

### 4.9. Analysis of ROS, Mitochondria, and Lysosomes

Cells were treated with the compounds for the indicated time, stained for 30 min with either 10 μg/mL JC-1 dye as mitochondrial potential indicator, LysoTracker Red (50 nM) as lysosomal stain, H_2_DCFDA (10 µM) as a total ROS indicator, or MitoSOX™ Red (5 µM) as mitochondrial ROS indicator, and were further analyzed flow cytometrically or microscopically. Mitochondrial potential loss is presented as mean percentage of cells with a decreased red to green JC-1 fluorescence intensity ratio [27]. For analysis of autophagy, HeLa cells expressing GFP-RFP-tagged LC3 were treated with chrysosplenol d or rapamycin for 4 h and formation of fluorescent LC3-positive autophagic punctae was assessed by fluorescent microscopy at 400× magnification. Nuclei were counterstained with DAPI.

### 4.10. Human Phospho-Kinase Array and Western Immunoblotting

Analysis of kinase activation upon treatment with chrysosplenol d and casticin was performed according to manufacturer′s instructions (Proteome Profiler Human Phospho-Kinase Array ARY003B, R&D Systems, Minneapolis, MN, USA). Cells were serum starved for 12 h followed by treatment with chrysosplenol d, casticin (both 30 µM), U0126 (10 µM), or 0.5% DMSO vehicle control for 3 h. After lysis and quantification of protein levels using a BCA assay (Thermo Fisher Scientific, Waltham, MA, USA), equal amounts of protein (300 µg) were incubated with membranes spotted with antibodies against various phosphorylated kinases. Membranes were analyzed using an Amersham^TM^ Imager 680 (GE Healthcare, Chicago, IL, USA). Alternatively, cells were treated with chrysosplenol d (10 µM), casticin (1 µM), or U0126 (10 µM) and whole cell lysates were separated by sodium dodecyl sulfate-polyacrylamide gel electrophoresis (SDS-PAGE) and electrophoretically transferred onto polyvinylidene fluoride membranes. Antibodies used were as follows: anti-ERK1/2, anti-AKT-1, anti-phospho-ERK1/2 (T202/Y402), anti-phospho-AKT (S473) (all from Cell Signaling Technology, Danvers, MA, USA), and anti-actin (Merck Millipore, Darmstadt, Germany). Proteins were visualized with above antibodies, detected with corresponding horseradish peroxidase-coupled secondary antibodies and ECL™ Prime Substrate (GE Healthcare) using an Amersham^TM^ imager 680. Autophagy was assessed by western immunoblot analysis of LC3II and p62 in whole cell lysates of MDA-MD-231 cells treated with vehicle or chrysosplenol d for 24 h. Antibodies were from Cell Signaling Technology. The anti-LC3 antibody used (#4108) recognizes preferentially the lipidated LC3II form.

### 4.11. Statistical Analysis

If not indicated otherwise, quantitative results are expressed as mean ± standard error of the mean (SEM) of at least three independent experiments. In case of two-group comparison and normally distributed data, analysis was done with the two-tailed Student´s t-test. Multigroup analysis was performed with the one-way analysis of variance or the Kruskal-Wallis test followed by the Newman-Keuls post hoc test. Correlations were investigated by the Pearson′s correlation test. For analysis, SigmaPlot software was used (Systat Software Inc., San Jose, CA, USA). Significance levels were set at * *p* < 0.05, ** *p* < 0.01, and *** *p* < 0.001.

## 5. Conclusions

The medicinal plant *Artemisia annua* is a source of antimalarial artemisinin. An artemisinin-free extract was toxic to cancer cells implicating existence of additional biologically active compounds. Chrysosplenol d and casticin, two flavonols identified, exhibit potential antitumor activity. Despite structural similarities, the flavonols target different signaling pathways. In TNBC with aberrant activation of the K-Ras/MAPK/ERK1/2 pathway, chrysosplenol d selectively augments phosphorylation of ERK1/2. Prolonged ERK1/2 activation may represent a novel approach for treatment of cancers with strong basal Ras/MAPK/ERK1/2 activation.

## Supplementary Materials

Supplementary materials can be found at https://www.mdpi.com/1422-0067/21/11/4090/s1. Figure S1. One dimensional 1H and 13C NMR spectra of chrysosplenol d. Figure S2. Two dimensional 1H and 13C NMR spectra of chrysosplenol d. Figure S3. ROS scavengers attenuate the chrysosplenol d-induced cytotoxicity. Figure S4. Akt activation is dispensable for chrysosplenol d-induced cytotoxicity. Figure S5. The MEK-inhibitor U0126 induces dissipation of the mitochondrial membrane potential (ΔΨm) in breast cancer cells and does not prevent ΔΨm loss induced by chrysosplenol d.

## Abbreviations

MDPI	Multidisciplinary Digital Publishing Institute
DOAJ	Directory of open access journals
TLA	Three letter acronym
AKT	Protein kinase B
AZKIM	Academic Center for Complementary and Integrative Medicine
BCI	(E)-2-benzylidene-3-(cyclohexylamino)-2,3-dihydro-1H-inden-1-one
BCL2	B-cell lymphoma 2
CAM	Chorioallantoic membrane
DMSO	Dimethyl sulfoxide
ERK	Extracellular signal-regulated kinase
ESI	Electrospray ionisation
FBS	Fetal bovine serum
ΔΨ_m_	Mitochondrial membrane potential
NMR	Nuclear magnetic resonance
GFP	Green fluorescent protein
GOF	Gain of function
GR_50_	Half-maximal growth rate inhibition
HPLC-DAD-MS	High-performance liquid chromatography–diode array & mass spectrometric detection
HPLC-MS/MS	High-performance liquid chromatography-tandem mass spectrometry
IC_50_	Half-maximal inhibitory concentration
LC3	Microtubule-associated proteins 1A/1B light chain 3B
LC3II	Lipid-modified form of microtubule-associated proteins 1A/1B light chain 3B
LOD	Limit of detection
LOQ	Limit of quantification
MEK	Mitogen-activated protein kinase kinase
MRM	Multiple-reaction monitoring
NSCLC	Non-small-cell lung carcinaoma
PBMC	Peripheral blood mononuclear cells
PI3K	Phosphoinositide 3-kinase
Ras	Rat sarcoma oncogen
ROS	Reactive oxygen species
TNBC	Triple negative breast cancer
XTT	2,3-bis-(2-methoxy-4-nitro-5-sulfophenyl)-2h-tetrazolium-5-carboxanilide
