# Supplementary material for: Chrysosplenol d, a Flavonol from Artemisia annua, Induces ERK1/2-Mediated Apoptosis in Triple Negative Human Breast Cancer Cells
Source: Int J Mol Sci. 2020 Jun 8;21(11):4090. doi: 10.3390/ijms21114090 (PMC7312517; doi:10.3390/ijms21114090)
Supplement: Supplementary file 1 [file ijms-21-04090-s001.pdf]

International Journal of *Molecular Sciences*

Supplementary Materials

Chrysosplenol D, a Flavonol from *Artemisia annua*, Induces ERK1/2-Mediated Apoptosis in Triple Negative Human Breast Cancer Cells

Sophia J. Lang [1], Michael Schmiech [1], Susanne Hafner [1], Christian Paetz [2], Katharina Werner [1], Menna El Gaafary [1,3], Christoph Q. Schmidt [1], Tatiana Syrovets [1,*,†], and Thomas Simmet [1,*,†]

[1] Institute of Pharmacology of Natural Products and Clinical Pharmacology, Ulm University, 89081 Ulm, Germany; sophia.lang@uni-ulm.de (S.J.L); michael.schmiech@uni-ulm.de (M.S.); susanne.hafner@uni-ulm.de (S.H.); katharina.werner@uni-ulm.de (K.W.); mennat_elgaafary@yahoo.com (M.E.G); christoph.schmidt@uni-ulm.de (C.Q.S.)

[2] Max Planck Institute for Chemical Ecology, 07745 Jena, Germany; cpaetz@ice.mpg.de[3] Department of Pharmacognosy, College of Pharmacy, Cairo University, Cairo 11562, Egypt

* Correspondence: tatiana.syrovets@uni-ulm.de (T.S.); thomas.simmet@uni-ulm.de (Th.S.); Tel.: +49-731-500-65604 (T.S.); +49-731-500-65600 (Th.S.)

† These authors contributed equally to this work

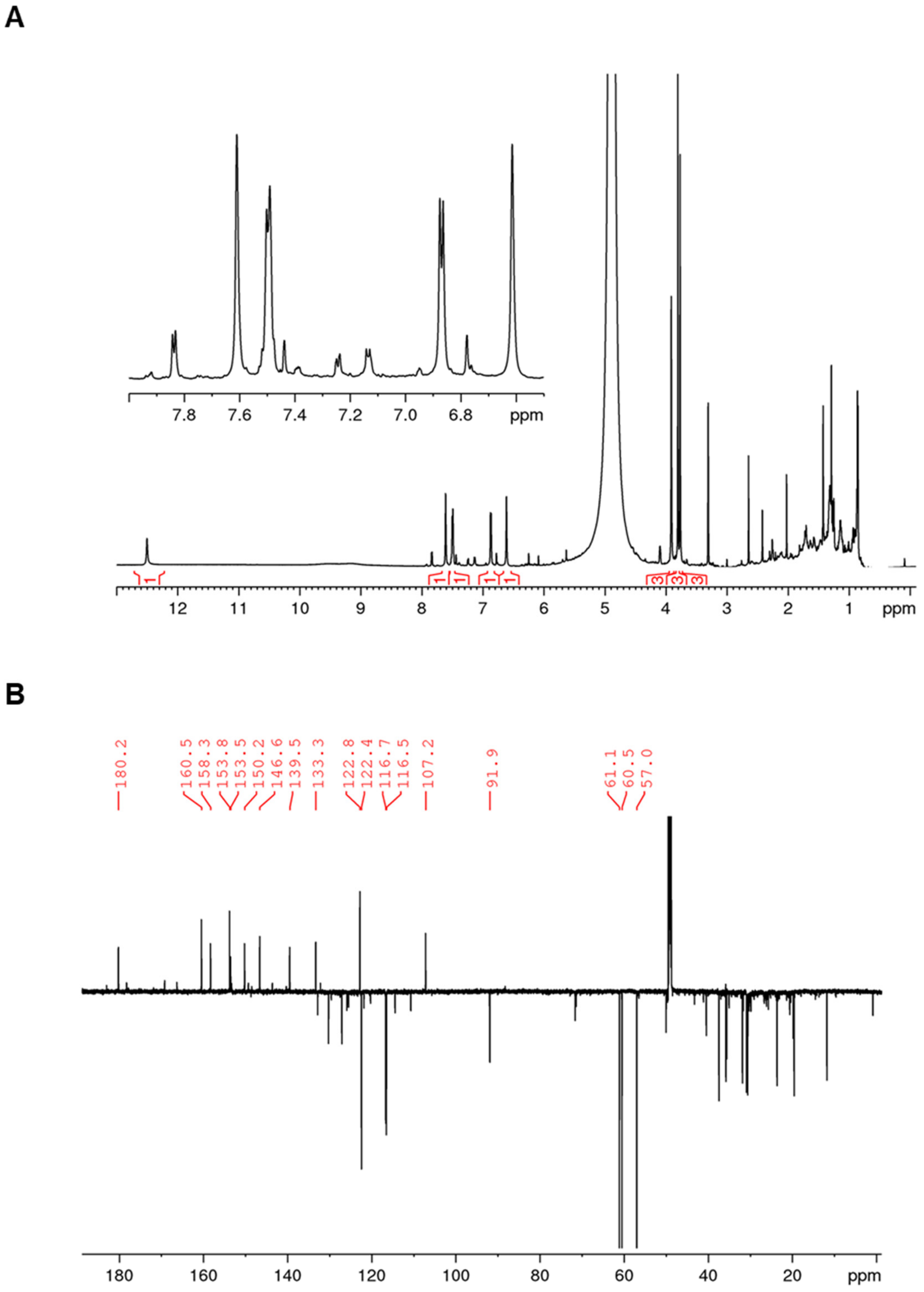

Figure S1. One dimensional ^{1}H and ^{13}C NMR spectra of chrysosplenol D. (**A**) ^{1}H NMR spectrum, 700 MHz, in MeOH-d3. (**B**) ^{13}C DEPTQ spectrum, 175 MHz, in MeOH-d3.

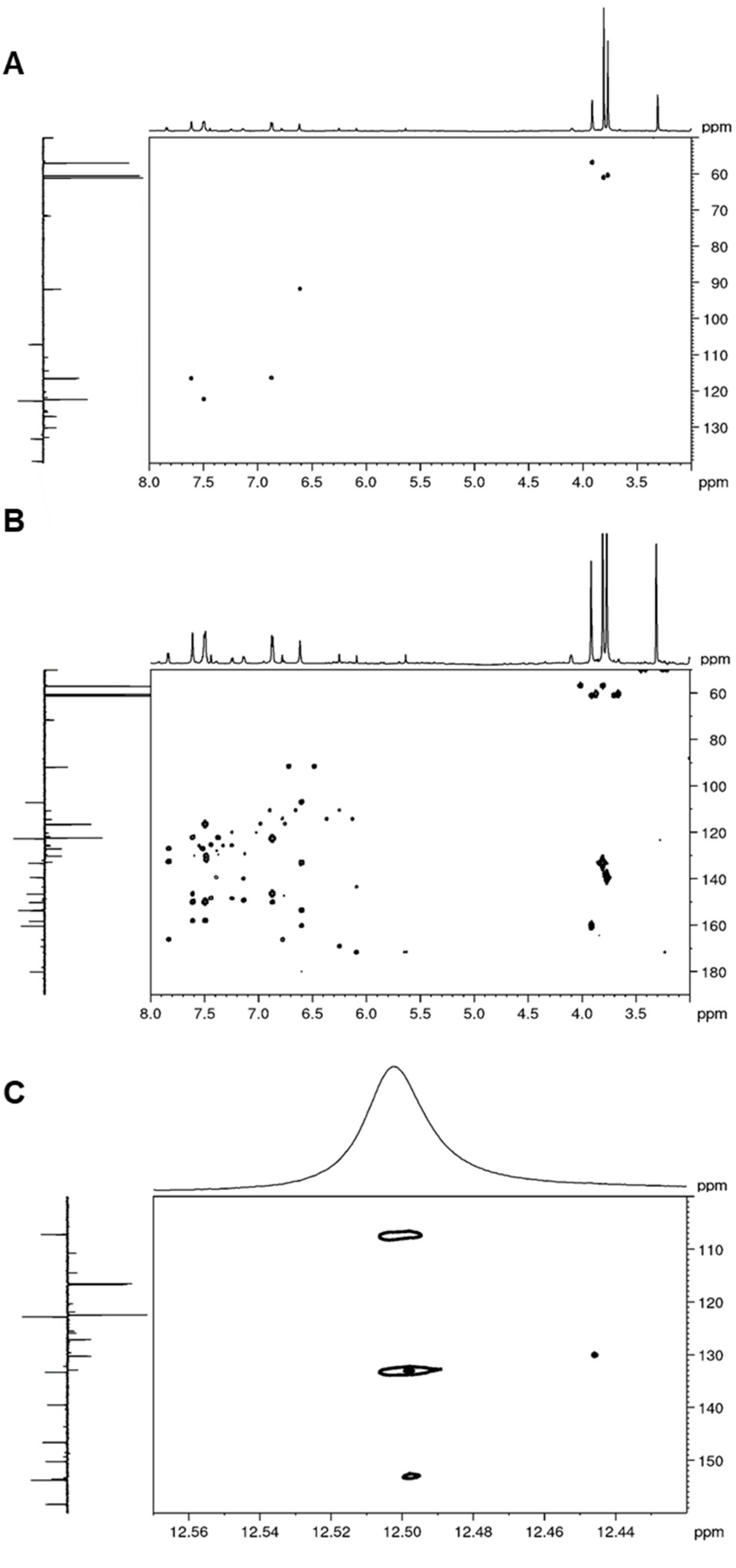

Figure S2. Two dimensional ^{1}H and ^{13}C NMR spectra of chrysosplenol D. (**A**) ^{1}H-^{13}C HSQC spectrum, 700/175 MHz, in MeOH-d3, level adjusted to main compound. (**B**) ^{1}H-^{13}C. HMBC spectrum, 700/175 MHz, in MeOH-d3, level adjusted to main compound. (**C**) Detail of ^{1}H-^{13}C HMBC spectrum, 700/175 MHz, in MeOH-d3, long-range ^{1}H-^{13}C correlations of OH at C-5.

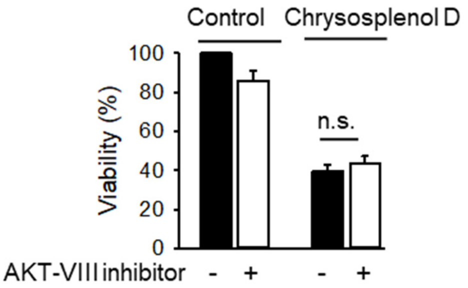

Figure S3. Akt activation is dispensable for chrysosplenol D-induced cytotoxicity. MDA-MB-231 cells were treated with an Akt activation inhibitor (AKT VIII inhibitor, Calbiochem, 0.2 μM) for 1 h and then with chrysosplenol D (10 μM) for 48 h. Cell viability was analyzed by using XTT. Data are mean ± SEM, n = 4, n.s. – non-significant.

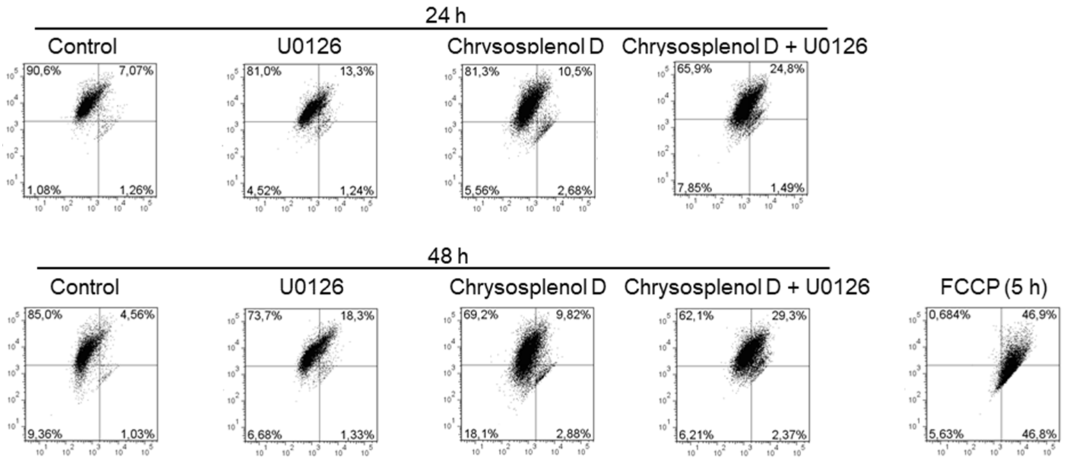

Figure S4. The MEK-inhibitor U0126 induces dissipation of the mitochondrial membrane potential ($\Delta\Psi_m$) in breast cancer cells and does not prevent $\Delta\Psi_m$ loss induced by chrysosplenol D. MDA-MB-231 cells were pretreated with the MEK-inhibitor U0126 (5 μM, 1 h) and treated with chrysosplenol D (10 μM) for 24 or 48 h. Loss of the mitochondrial membrane potential was analyzed by using flow cytometry after staining with JC-1. The uncoupling agent FCCP (carbonyl cyanide-4-(trifluoromethoxy)phenylhydrazone, 50 μM, 5 h) was used as positive control. Representative dot plots are shown.
